# Metastatic Crohn's Disease: An Approach to an Uncommon but Important Cutaneous Disorder

**DOI:** 10.1155/2017/8192150

**Published:** 2017-01-03

**Authors:** Babak Aberumand, Jessica Howard, John Howard

**Affiliations:** ^1^Schulich School of Medicine & Dentistry, Western University, London, ON, Canada; ^2^Department of Family Medicine, Schulich School of Medicine and Dentistry, Western University, London, ON, Canada; ^3^Departments of Medicine and Pediatrics, Schulich School of Medicine and Dentistry, Western University, London, ON, Canada

## Abstract

*Objective*. To provide physicians with a clinical approach to metastatic Crohn's disease (MCD).* Main Message*. Metastatic Crohn's disease, defined as skin lesions present in areas noncontiguous with the gastrointestinal tract, is the rarest cutaneous manifestation of Crohn's disease. MCD lesions vary in morphology and can arise anywhere on the skin. MCD presents equally in both sexes and across age groups. Cutaneous findings may precede, develop concurrently with, or follow gastrointestinal involvement. A detailed history and thorough physical examination including a full-skin exam may help to exclude other dermatoses, as MCD can mimic other common disorders. A biopsy is required for a definitive diagnosis. Treatment options for MCD remain underwhelming due to the lack of randomized control studies and varying responses of reported therapeutic methods. Topical, intralesional, and systemic corticosteroids, antibiotics, traditional immunosuppressants, and surgery have shown mixed results. Recently, biologics have shown promise, even with refractory cases of MCD.* Conclusion*. MCD is an important cutaneous manifestation of this inflammatory disorder. Although a rare entity, early recognition can provide opportunity for successful therapeutic intervention.

## 1. Case

**Figure 1:**
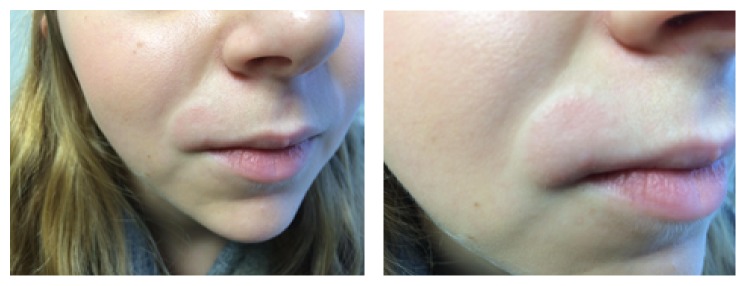
Patient presentation of two-centimeter, well-demarcated, pink-coloured oval plaque on the right cutaneous upper lip consistent with MCD.

A 20-year-old female with Crohn's disease presented to her family physician with a lesion on her right upper lip. It had been enlarging over the past two years, initially appearing after being treated with cefprozil for streptococcal sore throat. Her Crohn's disease was in remission, following four surgeries, and she was taking no medications. She was otherwise healthy. During the assessment, she stated that the lesion was usually pale pink, but on occasion became bright red. Upon skin examination, a two-centimeter, well-demarcated pink-coloured oval plaque was observed on her right cutaneous upper lip (Figure 1). A punch biopsy of the lesion was performed.

## 2. Introduction

In 1932, Dr. Burrill B. Crohn and his colleagues described a disease characterized by subacute or chronic necrotizing inflammation of the terminal ileum. They referred to this as regional ileitis [1]. It was soon discovered that this inflammatory disorder could affect any region of the gastrointestinal tract and was given the name Crohn's disease, in honour of Dr. Crohn [2].

In 2012, an estimated 129,000 Canadians suffered from Crohn's disease [3]. Hence, the inflammatory bowel disease is a common illness that gastroenterologists encounter. 22 to 44% of patients with Crohn's disease have cutaneous symptoms, making the skin the most common site of extraintestinal involvement [4, 5]. This association between the gut and skin can be classified into three broad groups described in Table 1.

In 1965, Parks et al. first described noncaseating granulomas at sites outside of the gastrointestinal tract. In 1970, Mountain coined the term MCD after encountering two further cases. Both cases demonstrated the same distinguishing pathologic findings of gastrointestinal Crohn's disease, but at sites distant from the gastrointestinal tract [6].

## 3. Clinical Presentation

The cutaneous manifestations of MCD have fairly variable morphology [10, 11]. When present in intertriginous areas, lesions tend to have an ulcerated appearance. Lesions on the extremities tend to be erythematous and sometimes painful. Other morphological descriptions include violaceous perifollicular papules and lichenoid papules found on the neck and lower limbs and erythematous plaques on the face and the extremities [10, 12]. The most common areas of involvement for MCD are the legs, vulva, penis, trunk, and face [11].

Other reported areas include the breast, nipple, ear, and umbilicus [13–16]. A predisposition of these lesions to moist environments such as intertriginous and flexural areas has been documented in the literature [17]. MCD affects both sexes and all ages equally [10, 12]. Children and adults often differ in clinical presentation [18, 19]. No clear correlation between the development of MCD and severity of underlying Crohn's has been established. In fact, the skin lesions may be present during, after, and in rare cases before the presence of the inflammatory bowel disease. The cutaneous findings tend to occur more often with Crohn's disease that involves the large bowel. This is different from gastrointestinal-only Crohn's disease where the small bowel, primarily the terminal ileum, is most commonly affected [11, 12, 17, 20–23].

## 4. Pathogenesis

Similar to Crohn's disease, the exact etiology of MCD remains unknown. However, two predominant theories have been suggested [12, 24]. One theory relies on the notion that the lesions are due to a granulomatous response involving perivascular localization of monocytes and epithelioid histiocytes to unknown antigens from the gastrointestinal tract carried through the circulatory system and eventually deposited within the skin [10, 25, 26]. The second theory suggests a granulomatous vasculitis picture secondary to a type IV hypersensitivity reaction in which sensitized T lymphocytes react to circulating antigens releasing various lymphokines and activating monocytes resulting in granulomatous damage of the vessel wall and inflammation [10, 26–28]. If the vascular changes are the trigger of MCD or the result of secondary changes remains to be determined [20, 29].

Another emerging hypothesis is that the cause of MCD may be more multifactorial involving a collaborative effort between immune mechanisms, alteration of enzymes, genetic factors, and bacterial Id reactions [10, 20]. However, no evidence of bacterial RNA has been uncovered in the cutaneous lesions of MCD to date [18, 30].

## 5. Histology

Microscopically, the dominant histological features of MCD are similar to the primary Crohn's bowel lesions [10, 26]. These include an inflammatory infiltrate commonly consisting of sterile noncaseating sarcoid-type granulomas, foreign body and Langhans giant cells, epithelioid histiocytes, and plasma cells surrounded by numerous lymphomononuclear cells found within the dermis and occasionally extending into the subcutis [8–10, 12, 20, 26]. Some of these granulomas are arranged perivascularly with normal vessel walls [12, 28, 31–33], whereas others exhibit a perivasculitic pattern, as they are associated with small- and medium-vessel vasculitis [12, 20, 21, 25, 27, 28, 34, 35]. The perivascular arrangement is attributed to the theory that MCD is the result of either deposition of immune complexes or circulating antigens within the skin [7, 28]. Other less common features that have been noted in the literature to be found in MCD include collagen degeneration known as necrobiosis [10, 17, 22, 35, 36], infiltrate rich in eosinophils with overlying ulceration in the epidermis [20, 28], edema of the dermis [37, 38], and lichenoid and granulomatous dermatitis [20].

## 6. Diagnosis, Differential Diagnosis, and Investigations

Metastatic Crohn's disease is known as a great mimicker and is often misdiagnosed [7, 12, 20, 31, 39]. The differential diagnosis is listed in Table 2. A detailed history and physical exam may help narrow the differential. However, a biopsy of the lesion is necessary in diagnosis of MCD. Other studies such as Periodic Acid-Schiff (PAS) staining, acid- and alcohol-fast bacilli testing, tissue cultures, and tuberculin skin test could also be done to exclude mimickers of MCD [2, 12, 19, 24, 39].

## 7. Treatment

Metastatic Crohn's disease can cause significant morbidity [17]. Although it has been reported that the cutaneous lesions of MCD can spontaneously resolve [10, 25, 28], the vast majority are persistent [22]. Unfortunately, there is no consensus on standard treatment for MCD as no clinical trials have been done to guide treatment [10, 17]. Several methods have been trialed varying in treatment length and response [18]. Topical treatments such as Milton's solution, Burrow's solution, potassium permanganate, sorbolene, lotio rubra, and antifungal creams (clotrimazole) and oral psoralen therapy with UV-A have not produced adequate results [2, 6, 10, 17, 24]. Conversely, improvement and resolution have been achieved with topical steroids such as betamethasone valerate 0.1% cream, betamethasone dipropionate 0.05% cream, and clobetasol propionate 0.05% cream applied two or three times per day [40–43]. Furthermore, oral prednisone or prednisolone 20–40 mg daily has demonstrated a favourable response [10, 11, 26–28]. In recalcitrant MCD lesions originally treated with prednisolone, adding sulfasalazine 3% ointment topically or sulfasalazine 2 to 4 gm/day systemically has been successful. The thought is that sulfasalazine plays a role in accelerating the healing of MCD lesions [10, 11, 22, 28]. Furthermore, metronidazole 800 mg to 1.2 gm daily has shown mixed results [2, 10, 24]. Other oral antibiotics such as cephalexin and ciprofloxacin have been infrequently used and have not proven to be beneficial [17, 24].

Surgical debridement, much like hyperbaric oxygen with concomitant metronidazole therapy, has been found to be effective in treating MCD in the perineal region [44, 45]. Surgical removal of the areas of intestine affected by Crohn's disease does not inevitably improve MCD or prevent its development [19, 46].

Cyclosporine 4 mg/kg/d or 250 mg BID [47–49], azathioprine 2 mg/kg [40], and 6-mercaptopurine have also shown some promise as most cases treated with each immunosuppressive agent demonstrated dramatic improvement [19]. Alternatively, if the use of either of those immunosuppressive drugs is not feasible, mycophenolate mofetil has also been shown to heal MCD skin lesions at a dose of 500 mg BID in a patient who was concomitantly taking 100 mg once daily of thalidomide [50].

Some patients have benefited from repeated curettage and simultaneous use of oral zinc sulfate for ulcerative-type of lesions [6]. Severe refractory MCD has been shown to respond to infliximab 400 mg infusions over a period of 7 weeks. Concomitant oral metronidazole and ciprofloxacin were administered to reduce the risk of the development of subcutaneous abscesses. In one case, methotrexate was also concurrently used with infliximab treatment [24, 51–54]. In patients who have had a prior infusion reaction to infliximab, certolizumab combined with methotrexate has recently been shown to resolve the cutaneous lesions [55]. Another biologic, adalimumab, has also shown efficacy in inducing and maintaining remission [56, 57]. In fact, adjunct usage of topical tacrolimus 0.1% twice daily with adalimumab has demonstrated improvement [58]. Individual usage of tacrolimus 0.1% OD has also been found to be safe and effective in treating MCD [59]. Other noteworthy efficacious treatment options for MCD include tetracycline hydrochloride ointment [60], mesalamine 800 mg three times daily with prednisone 80 mg/day [61], and intralesional triamcinolone [4]. Recurrence may take place with any of the above treatment modalities. Healed MCD lesions can leave behind hypertrophic scars; however these have been well treated with CO_2_ laser [11].

## 8. Revisiting the Case

**Figure 2:**
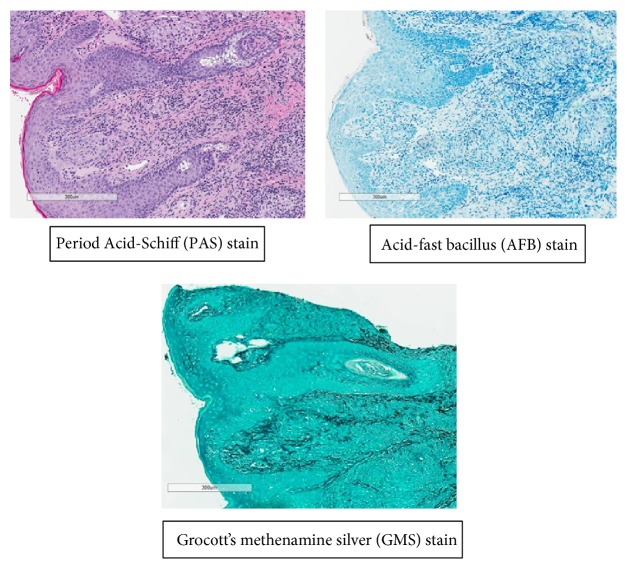
Histopathology findings of the patient's MCD lesion.

Biopsy results showed well-formed granulomas surrounded by mixed chronic inflammatory infiltrate of lymphocytes and plasma cells present throughout the dermis extending into the superficial fat. Focal spongiosis and exocytosis were also noted. Examination by polarization and stains for fungi and mycobacteria were negative (Figure 2). Given the clinical history and biopsy results, the diagnosis of MCD was made. Various treatment options from the use of topical to systemic medication were discussed with the patient. She opted for a conservative approach and a trial of tacrolimus 0.1% ointment BID was started. Marked improvement of the lesion was observed at her six-month follow-up appointment.

## 9. Conclusion

Although MCD remains a rare cutaneous manifestation of Crohn's disease, it is an important entity for gastroenterologists to be aware of, given the potential for misdiagnosis. This stems from the variability in morphologic presentation and similarities to other dermatoses. A detailed history and physical examination including a thorough skin exam should be conducted to help guide diagnostic possibilities. However, a biopsy is ultimately required for a definitive diagnosis. Treatment remains a challenge given the variable response to therapeutic options reported in the literature and the lack of a randomized control studies. Despite incidents of spontaneous resolution, the cutaneous lesions are often persistent and cause significant morbidity. Consequently, an early diagnosis makes successful therapeutic intervention more promising.

## Figures and Tables

**Table 1:** Classification of cutaneous manifestations of Crohn's disease.

Group	Type of lesions/diseases
(i) Direct extension from the bowel to the adjacent skin such as the perineal skin, stomal sites or lips [7]	(i) Fissures (ii) Fistulas (iii) Anal tags (iv) Oral involvement

(ii) Dermatoses with a strong association with Crohn's disease [2, 7, 8]	(i) Pyoderma gangrenosum (ii) Erythema nodosum (iii) Erythema multiforme (iv) Epidermolysis bullosa acquisita (v) Oral aphthae (vi) Acrodermatitis enteropathica (vii) Skin changes secondary to malabsorption (viii) Psoriasis (ix) Vitiligo (x) Cutaneous polyarteritis nodosa

(iii) Skin lesions at sites noncontiguous to gastrointestinal tract [9]	(i) Metastatic Crohn's disease

**Table 2:** Differential diagnosis of metastatic Crohn's disease [2, 10, 12, 18–20, 31, 39].

Granulomatous disorders	Nongranulomatous disorders
(i) Cutaneous sarcoidosis (ii) Tuberculosis (iii) Syphilis (iv) Mycobacterial infections (v) Actinomycosis (vi) Deep fungal infections (vii) Lymphogranuloma venereum (viii) Granuloma inguinale	(i) Hidradenitis suppurativa (ii) Pyoderma gangrenosum (iii) Impetigo (iv) Erythema nodosum (v) Factitial dermatitis from factitial injection of foreign substances (vi) Schistosomiasis (vii) Chronic lymphedema resulting from obstruction (viii) Erysipelas (ix) Chronic cellulitis (x) Foreign body reaction
